# The effect of increasing Women's autonomy on primary and repeated caesarean sections in Brazil

**DOI:** 10.1002/hec.4522

**Published:** 2022-05-23

**Authors:** Victor Hugo de Oliveira, Ines Lee, Climent Quintana‐Domeque

**Affiliations:** ^1^ Norwich Medical School University of East Anglia Norwich UK; ^2^ Faculty of Economics University of Cambridge Cambridge UK; ^3^ Department of Economics Business School University of Exeter Exeter UK

**Keywords:** caesarian sections, natural experiment, policy change, synthetic control

## Abstract

Caesarean section (C‐section) rates continue to rise globally. Yet, there is little consensus about the key determinants of rising C‐section rates and the sources of variation in C‐section rates across the world. While C‐sections can save lives when medically justified, unnecessary surgical procedures can be harmful for women and babies. We show that a state‐wide law passed in São Paulo (Brazil), which increased women's autonomy to choose to deliver via C‐section even when not medically necessary, is associated with a 3% increase in overall C‐section rates. This association was driven by a 5% increase in primary C‐sections, rather than repeated C‐sections. Since the law emphasizes women's autonomy, these results are consistent with mothers' demand being an important contributor to high C‐section rates in this context.

## 1 INTRODUCTION

The percentage of babies born via Caesarean section (C‐section) has increased dramatically in recent decades. While C‐sections can be life‐saving when medically justified, recent evidence suggests that C‐section rates up to and above 10%–19% are not associated with decreases in neonatal or maternal mortality rates (Molina et al., 2015; WHO, 2015). C‐section rates vary widely across countries, from 1.4% in Chad to over 55% in Brazil (Betrán et al., 2016), yet there is little consensus about the key determinants of C‐sections over vaginal births (Baicker et al., 2006; MacDorman et al., 2008).

We investigate the impact of a state‐wide law that increases women's autonomy to choose their method of delivery on overall, primary, and repeated C‐section rates. This law (Law 17,137) was passed on August 23, 2019 in São Paulo (Brazil) and allows pregnant women from the 39^th^ week of gestation to choose to deliver via C‐section in public hospitals even if this procedure is not medically necessary (see Online Appendix A). Law 17,137 was inspired by Resolution 2144 passed by the Federal Council of Medicine which supports women's right to choose CS (C‐section) delivery in elective cases (see Online Appendix B). Lawmakers' rationale for implementing Law 17,137 is described in draft law 435/2019 (see Online Appendix C). In July 2020, Law 17,137 was declared unconstitutional by the São Paulo's Court due to the perceived conflict with the Constitutional Law (see Online Appendix D). Online Appendix E provides a brief description of the healthcare system in Brazil and its differences across states.

**FIGURE 1:**
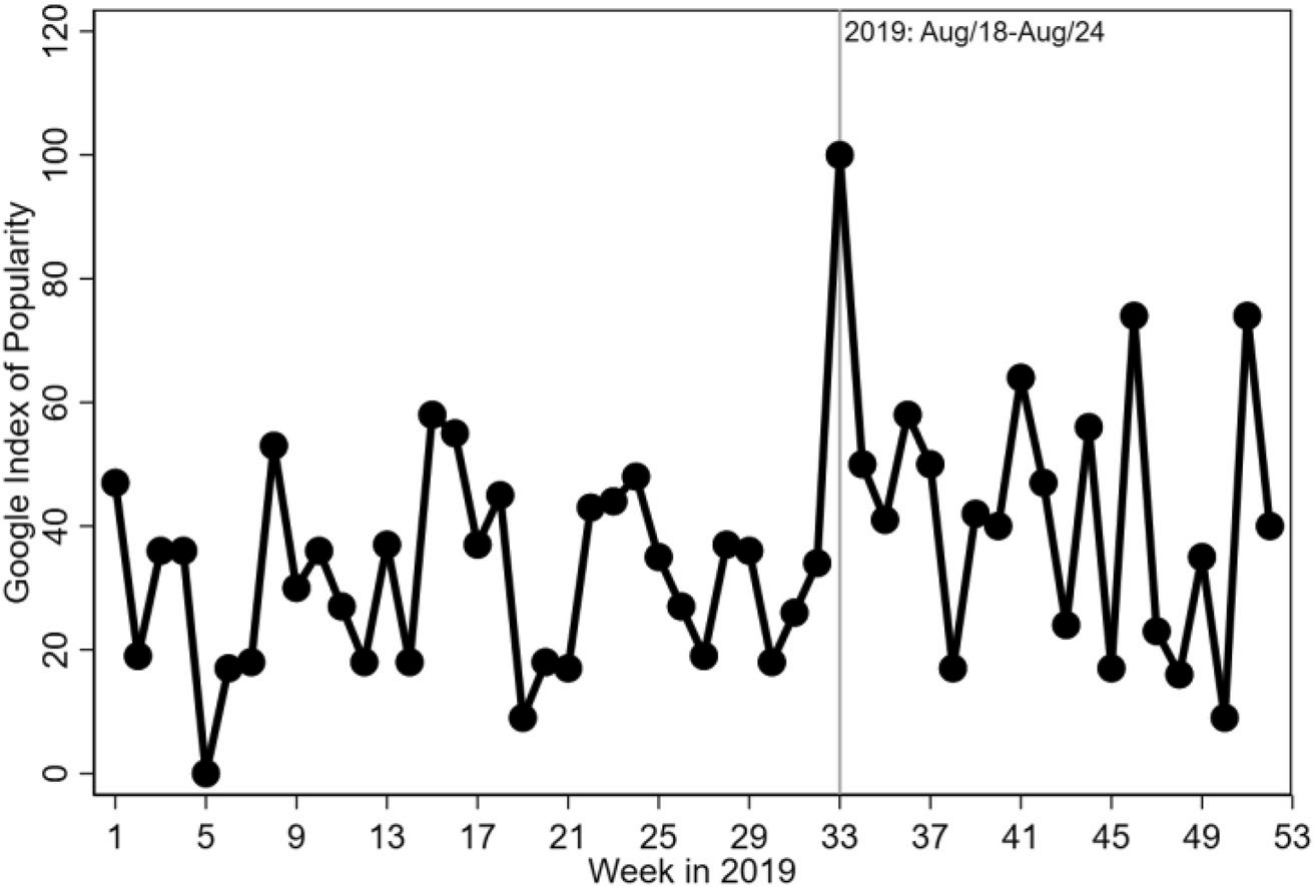
Google trends of the term “Parto Cesárea” (Caesarean section) in São Paulo. *Source*: Google trends

Data from Google Trends indicate awareness of Law 17,317 at the time of its announcement. Figure 1 shows that the index of popularity of the term “Parto Cesárea” (Caesarean section in Portuguese) in São Paulo doubled in the week of 18‐24 August compared to the previous week, consistent with the timing of media attention on this law and the publication of the law in São Paulo's official gazette on August 24, 2019.

## 2 MATERIALS AND METHODS

This study uses all recorded live births (8,730,318) in Brazil from January 1, 2017 to December 31, 2019 from the Brazilian Registry of Live Births (SINASC, https://datasus.saude.gov.br). Our main outcomes of interest are the number of overall, primary, and repeated C‐sections per 100 live births in each state at a bimonthly frequency (27 states, 18 periods, *n* = 486). Overall C‐sections were identified by a variable denoting the type of birth (vaginal or C‐section). Among those delivering by C‐section, primary (no previous) and repeated (previous) C‐sections were identified by a variable denoting the previous number of C‐sections. Hence, for a given period of 2 months, the number of live births is divided into vaginal and C‐section deliveries.

We use the synthetic control method (SCM) (Abadie, 2021; Abadie et al., 2010; Abadie & Gardeazabal, 2003) to assess the effect of Law 17,137 on overall, primary, and repeated C‐section rates. Standard differences‐in‐differences yield similar estimates, but we prefer using the SCM for reasons outlined in Online Appendix F. We compare the bimonthly evolution of C‐section rates in São Paulo to a weighted average of bimonthly C‐section rates among the other Brazilian states (synthetic counterpart), where the weights are chosen to match the trajectory of outcomes in São Paulo in the pre‐law period. In Online Appendix G, we provide the weights used to construct the synthetic control. To construct the synthetic São Paulo, we match on the respective outcome (overall, primary, or repeated C‐section) for 15 bimonthly periods prior to the implementation of the law (Jan‐Feb 2017 to May‐June 2019; Ferman et al., 2020) with one validation period (July‐August 2019). Our findings are robust to increasing the number of validation periods (Online Appendix H).

The estimated effect of the law is the difference between C‐section rates in São Paulo and its synthetic counterpart after the passage of the law (August 2019). Inference was conducted by comparing the estimated effect of the law in São Paulo with the distribution of estimated effects for selected placebo states (states where the law was not implemented). The selected placebo states are those for which the Root Mean Square Prediction Error is no more than five times that of São Paulo. We use 2‐sided *p*‐values and *p* < 0.05. We conduct permutation tests under the hypothesis that in the absence of the law the estimated effect of the law in São Paulo is not expected to be large relative to the distribution of estimated effects for selected placebo states.

The 2‐sided *p*‐value corresponding to this test is provided by the following equation (Cavallo et al., 2013; Galiani & Quistorff, 2017):

$$p=\mathrm{Pr}\left(\left|{\hat{\alpha }}_{1t}^{PL}\right|\geq \left|{\hat{a}}_{1t}\right|\right)=\frac{\sum_{j\neq 1}1\left(\left|{\hat{a}}_{jt}\right|\geq \left|{\hat{a}}_{1t}\right|\right)}{J},$$

where ${\hat{a}}_{1t}$ is the estimated effect for São Paulo (denoted by subscript 1) in a particular post‐law period t (e.g., September‐October 2019), jisthe *j*th selected placebo state, ${\hat{\alpha }}_{1t}^{PL}=\left\{{\hat{a}}_{jt}:j\neq 1\right\}$ is the distribution of corresponding estimated effects for any of the selected placebo states in the same period t, and J is the number of selected placebo states. Note that if there are no selected placebo states that have an estimated effect greater than the estimate for São Paulo, then $\sum_{j\neq 1}1\left(\left|{\hat{a}}_{jt}\right|\geq \left|{\hat{a}}_{1t}\right|\right)=0$ and the *p*‐value is equal to zero.

Under random assignment of the law, the *p*‐values from this permutation test have the same interpretation as in a classical randomization test. Under non‐random assignment of the law, which is more likely in our setting, *p*‐values can be interpreted as the proportion of selected placebo states that have an estimated effect at least as large as that of São Paulo. Analyses were conducted with Stata statistical software version 17 using the *Synth_Runner* package (Galiani & Quistorff, 2017).

The data and code for this analysis are available at https://doi.org/10.7910/DVN/W8SADO.

## 3 RESULTS

**FIGURE 2:**
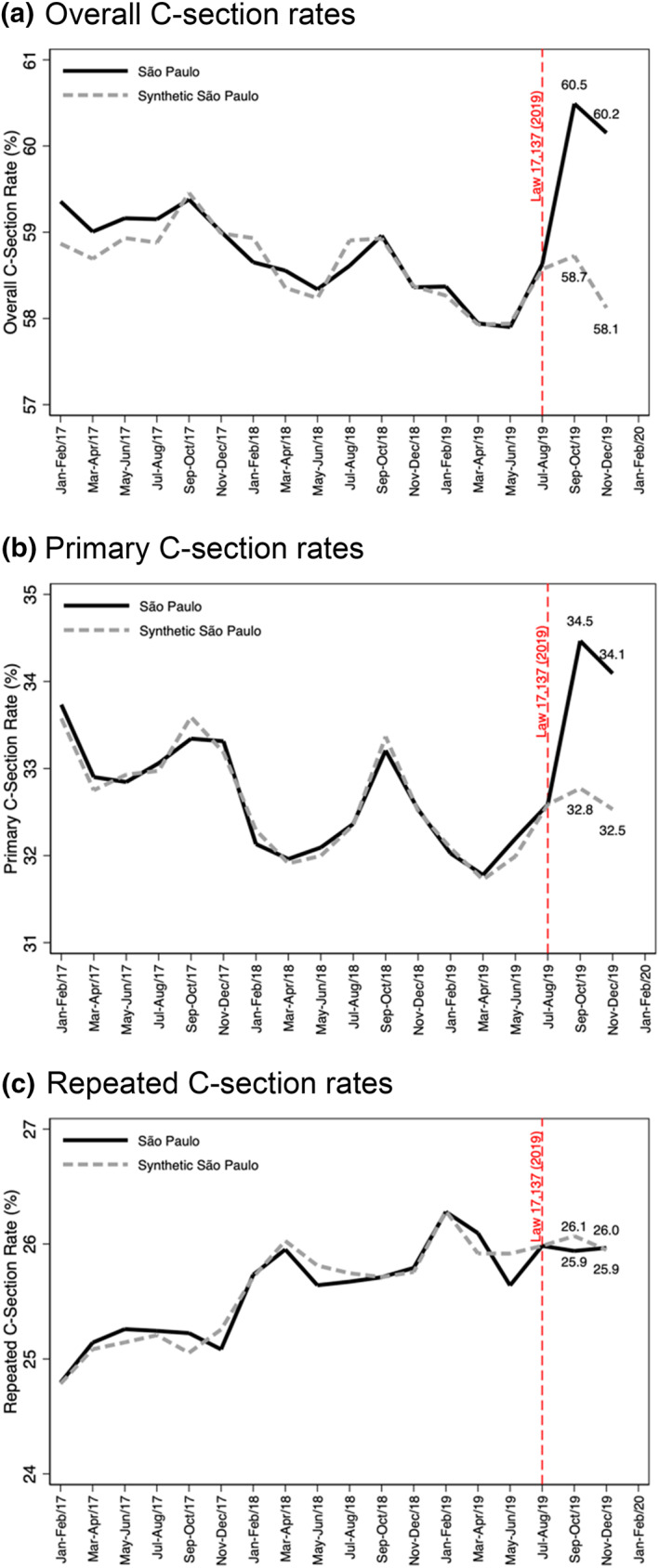
Overall (a), Primary (b), and Repeated (c) C‐section rates in São Paulo (bold line) compared to synthetic São Paulo (dashed line). The vertical dashed line denotes when Law 17,137 was implemented

Figure 2 displays the overall (A), primary (B), and repeated (C) C‐section rates in São Paulo and its synthetic counterpart between 2017 and 2019. Raw trends show a sharp increase in overall C‐sections after the passing of Law 17,137 (Online Appendix I). The solid bold line in Figure 2 is the bimonthly trend in the number of overall (A), primary (B), and repeated (C) C‐sections per 100 live births in São Paulo between January 2017 and December 2019. The solid dashed line is the bimonthly trend in the same outcomes in the synthetic counterpart. The vertical dashed line denotes when Law 17,137 was implemented.

In the pre‐law period (January 2017‐July 2019), the evolution of C‐Section rates in synthetic São Paulo closely tracks the trajectory of C‐Section rates in São Paulo. In September‐October 2019, overall C‐Section rates increased by 3.0% (*p* < 0.001) and primary C‐Section rates increased by 5.2% (*p* < 0.001). In November‐December 2019, overall C‐Section rates increased by 3.5% (*p* < 0.001) and primary C‐Section rates increased by 4.8% (*p* < 0.001). In contrast there was no associated change in repeated C‐Section rates in São Paulo either in September‐October 2019 (0.0%, *p* = 0.706) or in November‐December 2019 (0.0%, *p* = 0.941).

Additionally, we find that the increase in C‐section rates was higher among low‐educated, young, and unmarried mothers (Online Appendix J) and that Law 17,137 did not have any effects on fertility (Online Appendix K). Moreover, the effects of Law 17,137 are similar in areas with below‐ and above‐median availability of obstetricians and surgical obstetric beds, suggesting a limited role of supply factors (Online Appendix L). We are unable to detect any effects of Law 17,137 on infant health outcomes, though this analysis has limitations (Online Appendix M). We do not detect any effects when using a placebo policy implementation date (Online Appendix N). Moreover, excluding the four states that share borders with São Paulo, we find virtually the same effects (Online Appendix O). Finally, to benchmark our results, we provide a summary of quasi‐experimental studies (Amaral‐Garcia et al., 2021; Borra et al., 2019; Currie & MacLeod, 2008; de Elejalde & Giolito, 2021; Foo et al., 2017; Mühlrad, 2022) that examine other factors that influence C‐section rates (Online Appendix P).

## 4 DISCUSSION

The passage of Law 17,137 was associated with an increased in the primary, but not repeated, C‐section rate in São Paulo. This finding is notable given already high C‐section rates in Brazil and the likelihood of primary C‐sections resulting in future births being born via C‐sections (MacDorman et al., 2008). Since the law emphasizes women's autonomy (see Online Appendices A‐C) and since the effects of the law were similar across health regions with different availability of obstetricians and surgical obstetric beds (see Online Appendix L), these results suggest that mothers' demand is an important contributor to high C‐section rates in this context.

## 5 CONCLUSION

We provide the first step in documenting the effects of Law 17,137 on primary and repeated C‐section rates. This law and similar ones remain a contested issue in Brazil. While Law 17,137 was found unconstitutional and repealed by the Justice Court of São Paulo in 2020, a similar law ensuring women's choice over delivery methods is being currently discussed in the national legislative body of Brazil. Since method of delivery can have both short‐ and long‐term effects among mothers and children (Costa‐Ramón et al., 2020), policymakers may benefit from evidence on the effects of such laws. Future research and data collection efforts are needed to investigate the effects of the law on the choice of public versus private hospitals, as well as the effects of the documented increase in C‐section rates on maternal and neonatal mortality rates, fertility patterns 9 months later, and repeated C‐sections rates in the future (due to the health risks of vaginal births after caesarean).

## CONFLICT OF INTEREST

No conflict of interest disclosures were reported.

## Supporting information

Supporting Information S1

## Data Availability

The Brazilian Registry of Live Births are publicly available at https://datasus.saude.gov.br/. The data and code for the analysis in this study are publicly available at https://doi.org/10.7910/DVN/W8SADO.
