# Supplementary material for: The effect of increasing Women's autonomy on primary and repeated caesarean sections in Brazil
Source: Health Econ. 2022 May 23;31(8):1800–4. doi: 10.1002/hec.4522 (PMC9545260; doi:10.1002/hec.4522)
Supplement: Supplementary file 1 — Supporting Information S1 [file HEC-31-1800-s001.pdf]

The Effect of Increasing Women's Autonomy on Primary and Repeated Caesarean Sections in Brazil

Victor Hugo de Oliveira

Ines Lee

Climent Quintana-Domeque

Supporting Information S1 (Online Appendix)

This document contains supplementary material.

A. Additional Information on Law 17,137

On 23 August 2019 the Assembleia Legislativa do Estado de São Paulo passed law 17,137. The law outlines the follow clauses:

1. The pregnant woman has the right to an elective caesarean section (C-section) and must be respected in her autonomy.
2. The elective C-section will only be performed in pregnant women in the 39th week of gestation or above, after being made aware and informed about the benefits of vaginal delivery and the risks of successive C-sections.
3. The pregnant woman who chooses to have her child by vaginal delivery, presenting clinical conditions for this, must also be respected in her autonomy. The right to analgesia is guaranteed.
4. If the pregnant woman's option for C-section is not observed, the doctor will be obliged to record the reasons in the medical record.
5. In maternity wards and hospitals, a plaque with the following words will be affixed: "It is the right of the pregnant woman to choose either a vaginal delivery or a C-section, from the 39th week of gestation."
6. The doctor can always, in disagreement with the option made by the pregnant woman, refer her to another professional.
7. Expenses, arising from the implementation of this law, will be borne by own budget allocations, supplemented if necessary.

B. Additional Information on Resolution 2,144/2016

Law 17,137 was inspired by Resolution 2,144/2016, passed by the Federal Council of Medicine on 17 March 2016. This Resolution establishes that:

- Act 1: It is the right of the pregnant woman, in elective situations, to opt for a C-section, guaranteed by her autonomy, if she has received all the detailed information about the vaginal and C-section deliveries, their respective benefits and risks. The decision must be registered in a free and informed consent form, elaborated in an easily understood language, respecting the pregnant woman's sociocultural characteristics.
- Act 2: To guarantee the safety of the foetus, the C-section at the request of the pregnant woman, in situations of usual risk, can only be performed after the 39th week of pregnancy and must be registered in the medical record.
- Act 3: It is ethical for a doctor to perform a C-section on request, and if there is a disagreement between the medical decision and the pregnant woman's wishes, the doctor may claim her right to professional autonomy and, in these cases, refer the pregnant woman to another professional.

C. Additional Information on Draft Law 435/2019

Lawmakers' rationale for implementing Law 17,137 is described in draft law 435/2019. One of the important motivations of the law is captured by the views in the debate "Vaginal versus Caesarean Delivery". The key points discussed in this debate include:

- Women must have their autonomy respected when choosing method of delivery.

- In public healthcare systems, pregnant women can be subject to obstetric violence during vaginal delivery (e.g., the long length of delivery and labour pain). Caesarean deliveries may help overcome these issues.
- There is insufficient scientific evidence on the relationship between caesarean deliveries and maternal and infant deaths.

D. Additional Information on the Unconstitutionality of Law 17,317

In July 2020, Law 17,137 was declared unconstitutional by the São Paulo's Court due to the perceived conflict with the Constitutional Law. The Constitution of the Federative Republic of Brazil establishes that women can deliver their babies by caesarean section when medically justified. However, Law 17,137 allowed women to deliver babies via caesarean regardless of medical reasons. Moreover, the cost of caesarean sections in public hospitals is covered by the federal government, as all the costs of public health hospitals in Brazil. The Association of Obstetricians and Gynaecologists of São Paulo pointed out several shortcomings in Law 17,137 to the State Court. First, it is not clear which type of caesarean is covered by the law (e.g., elective, urgency, scheduled). Second, the motivation of the law provides no suitable scientific evidence to support the benefits to maternal and infant health. Third, there is insufficient evidence on the impact of the draft law on maternal and infant health.

E. Healthcare system in Brazil and overall C-section rates in 2018

The 1988 Constitution of the Federative Republic of Brazil establishes health as a right for all (therefore universal) and a duty of the State, to be guaranteed by social and economic policies (consistent with the founding mandate of the WHO) and by universal and equal access to health actions and services.[1] Prenatal and childbirth care are healthcare services provided by the Unified Health Service (Sistema Único de Saúde). In 2007, the Ministry of Health created the Table of Procedures, Medicines, Orthotics/Prostheses and Special Materials (Tabela de Procedimentos, Medicamentos, Órteses/Próteses e Materiais Especiais) to guide states and municipalities with respect to the budgetary and financial repercussions of outpatient and hospital production. This system provides values for hospital admissions according to the adopted medical procedures.

The reference value for a medical procedure is divided into hospital services and professional services. The value of hospital services includes per diems, room rates, food, hygiene, patient support staff in bed, materials, medicines and Auxiliary Services for Diagnosis and Therapy (ASDT), except special medicines and special ASDT. The values of professional services correspond to the fraction of professionals (doctors, dentists and obstetric nurses) who worked during the hospitalization. The outpatient service includes outpatient permanence fee, professional services, materials, medication, and support. This system does not differentiate reference values by state or geographical region. Therefore, the financial repercussion of a C-section or a vaginal delivery in the Unified Health System should be similar across states and municipalities, given the same involved inputs. Using cost data from three public maternity hospitals (two in Rio de Janeiro and one in Belo Horizonte), Entringer et al. (2018) measure the direct costs of elective caesarean and vaginal delivery with human resources, hospital inputs, and capital and administrative resources. The authors find that vaginal delivery was more

[1] Article 196. Health is a right of all and a duty of the State and shall be guaranteed by means of social and economic policies aimed at reducing the risk of illness and other hazards and at the universal and equal access to actions and services for its promotion, protection and recovery.

efficient for primiparous women, at lower cost (BRL 1,709.58; USD 518.05) than caesarean (BRL 2,245.86; USD 680.56).

Despite the lack of studies about regional disparities regarding cost-effectiveness of caesarean and vaginal deliveries, the public health literature has documented regional disparities in the provision of healthcare services across Brazilian regions (Massuda et al. 2018). Such disparities have been observed in the coverage and quality of birth care service. The coverage of health facilities is much larger in the Southeast and South regions, which is associated with high C-section rates in the corresponding regions compared to the rest of the country (Barros et al., 2015). The literature also shows that most maternity wards across the country have a low rate of adequacy that can affect the quality of labour and birth care, especially in the North and Northeast regions (de Azevedo Bittencourt et al. 2016). Coverage and quality of the prenatal care is worse in the North and Northeast region (Leal et al. 2020). On the other hand, interventions during labour (e.g. peripheral venous catheter, oxytocin drip, spinal/epidural analgesia, and amniotomy) are more frequent in the Southeast and South regions of the country (Leal et al. 2014).

Figure E1 displays the overall C-section rates across Brazilian states in 2018.

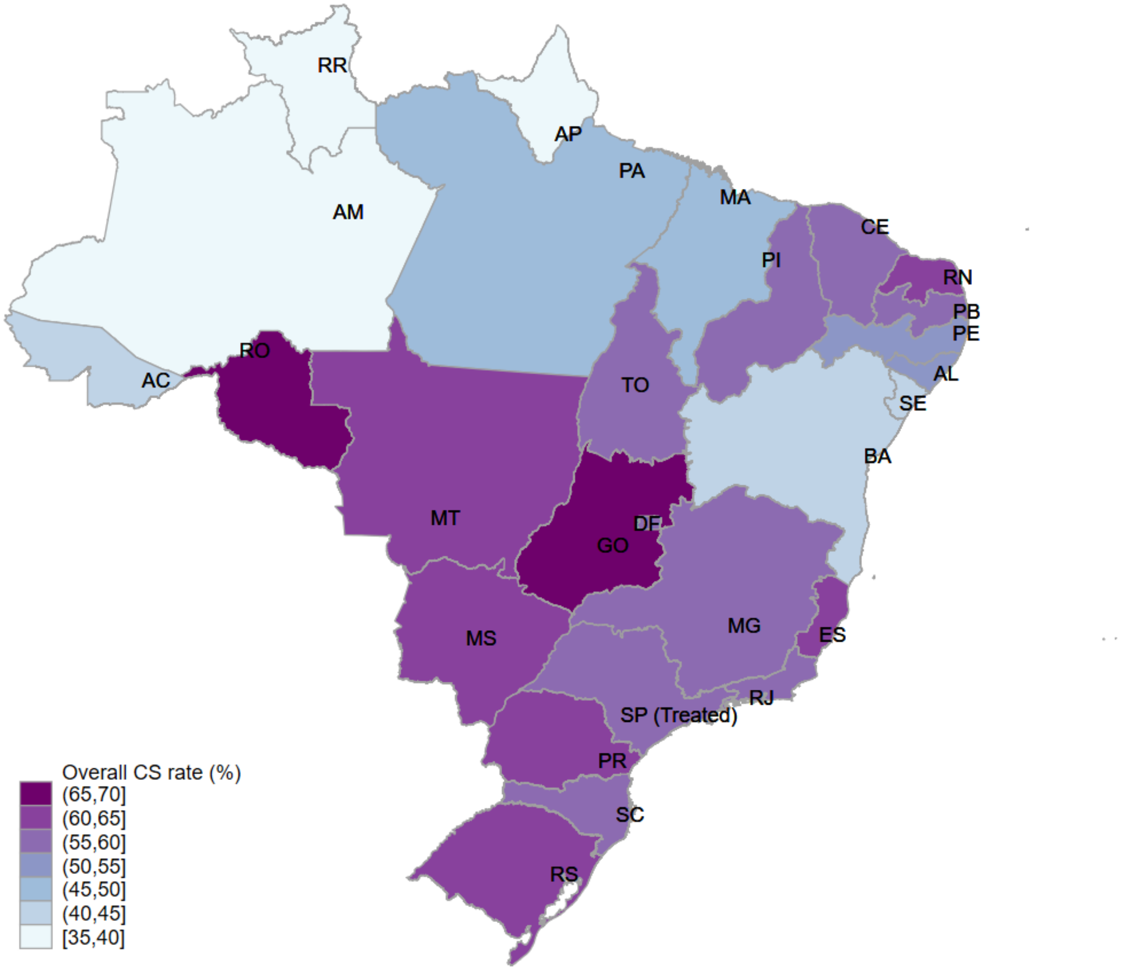

Figure E1: Map of overall C-section rates by state in 2018. See Table S2 for the list of states.

F. Standard 2-by-2 difference-in-differences point estimates

Table S1 reports the overall CS (C-section) rate in the state of São Paulo (SP) and the mean of overall CS rate for the rest of Brazil (RB). Using the standard 2-by-2 difference-in-differences (DiD) method, we compute an increase of 0.9 percentage point (pp) in the overall CS rate due to the introduction of the law in September-October 2019 and an increase of 1.1 pp in November-December 2019.

Table S1: Standard DiD (2x2) for the Overall CS Rate

	Rest of Brazil (RB)	São Paulo (SP)	*Difference (SP-RB)*
Sep-Oct/2018 (Before)	54.4%	59.0%	*4.6pp*
Sep-Oct/2019 (After)	55.0%	60.5%	*5.5pp*
Difference (After-Before)	*0.6pp*	*1.5pp*	**0.9pp**
Nov-Dec/2018 (Before)	54.0%	58.4%	*4.4pp*
Nov-Dec/2019 (After)	54.7%	60.2%	*5.5pp*
Difference (After-Before)	*0.7pp*	*1.8pp*	**1.1pp**

The standard 2-by-2 DiD point estimates are comparable to those using the synthetic control method (SCM). However, we use the SCM approach in the main analysis for two main reasons:

- First, as highlighted by Abadie (2021), the SCM is appropriate to estimate the effects of interventions that are implemented at an aggregate level affecting a small number of large units (such as a cities, regions, or countries), on some aggregate outcome of interest. In these setups with large control groups and small treatment groups, there is a large imbalance in sample size between groups which may affect statistical inference. As our setting involves an intervention affecting only one aggregate unit (the state of São Paulo), this makes SCM the most suitable approach for our setup.
- Second, sometimes the standard DiD is not enough and needs to be extended to include higher-order differences (de Oliveira et al. 2021). Indeed, there are many degrees of freedom when trying to find a control state and using difference-in-differences methods (including how to control for group-specific trends). SCM methods help us restrict the number of degrees of freedom and limit the scope for cherry-picking, at least ex ante (Ferman et al. 2020).

G. Weights to construct the synthetic control group

Table S2 below provides the weights used to construct the synthetic control for each of our main outcomes of interest.

Table S2: Weights to construct the synthetic control

State	**Overall CS**	**Primary CS**	**Repeated CS**
Rondônia (RR)	0.106	0.028	0
Acre (AC)	0	0.025	0.064
Amazonas (AM)	0	0	0
Roraima (RR)	0.025	0	0
Pará (PA)	0	0	0
Amapá (AP)	0	0	0
Tocantins (TO)	0	0	0
Maranhão (MA)	0	0.039	0
Piauí (PI)	0	0	0
Ceará (CE)	0	0.007	0
Rio Grande do Norte (RN)	0	0.041	0
Paraíba (PB)	0	0	0.021
Pernambuco (PE)	0	0	0.089
Alagoas (AL)	0.064	0.103	0.023
Sergipe (SE)	0	0	0.019
Bahia (BA)	0	0	0
Minas Gerais (MG)	0	0	0
Espírito Santo (ES)	0.180	0	0.134
Rio de Janeiro (RJ)	0.024	0	0.047
Paraná (PR)	0.093	0	0.192
Santa Catarina (SC)	0.287	0.170	0
Rio Grande do Sul (RS)	0	0.299	0
Mato Grosso do Sul (MS)	0	0.288	0.131
Mato Grosso (MT)	0	0	0
Goiás (GO)	0.220	0	0.089
Distrito Federal (DF)	0	0	0.191

It is worth noting that states with higher weights in the synthetic control group are, in general, states in the Southeast, South and Centre-West regions. This means that the donor pool used to generate the Synthetic São Paulo captures the regional disparities discussed in Appendix E.

Figure G1 displays the geographical distribution of weights to construct the synthetic control when studying overall C-section rates.

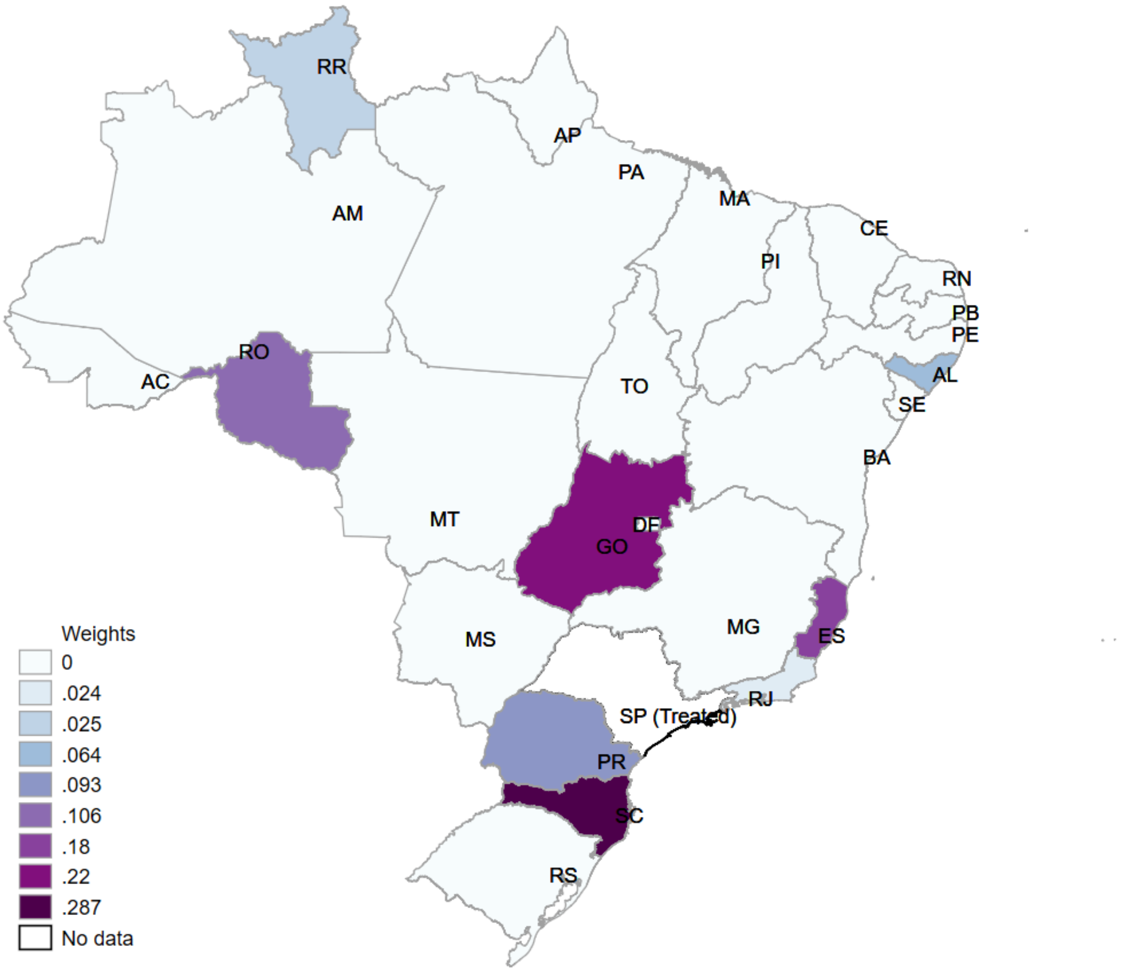

Figure G1: Map of the states that contribute to the synthetic control when studying the effect on the overall C-section rate. See Table S2 for the list of states.

H. SCM with more than one validation period

The existing literature on SCM suggests that the lengths of the training and validation periods depend on application-specific factors such as the extent of data availability on outcomes in the pre- and post-intervention period (Abadie 2021). In the research letter, we chose one validation period given the short time period that we are looking at (2 years at a bi-monthly frequency). There is a trade-off between using data points for validation and for prediction. By increasing the number of periods for validation, we lose information that can be used to predict the outcome of synthetic São Paulo.

In Table S3, we demonstrate that our results are robust to increasing the number of validation period by varying the number of validation periods from 2 to 4. The results are very similar to those in the research letter where 1 validation period is used.

Table S3

Panel A: Estimated overall CS rates for synthetic São Paulo using different number of validation periods

Num. validation periods	Estimated CS rate (Sep/Oct)	Estimated CS rate (Nov/Dec)
2	58.77%	58.17%
3	58.78%	58.18%
4	58.72%	58.12%
**Panel B: Estimated effects using different number of validation periods**		
Num. validation periods	Estimated Effect (Sep/Oct)	Estimated Effect (Nov/Dec)
2	1.72pp	1.98pp
3	1.71pp	1.97pp
4	1.77pp	2.03pp

I. Raw trends data on C-sections

Figure S1 shows that despite the seasonality in CS rates, there is a sharp increase in the overall CS rate after the passing of the law in São Paulo (vertical dashed red line). We do not see the same pattern in the CS rate for the rest of Brazil. Figures S2 and S3 show that this increase is driven by increases in primary CS rates rather than repeated CS rates.

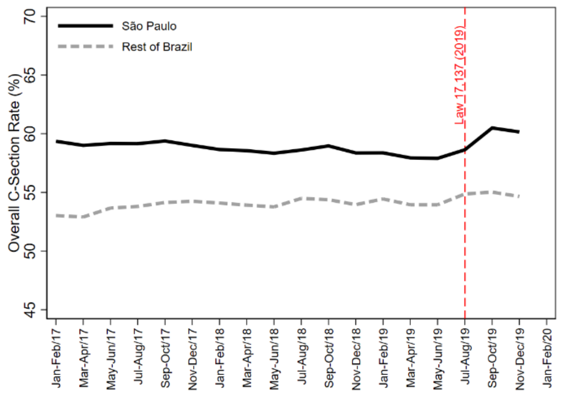

Figure S1: Raw bimonthly time-trend of Overall CS – São Paulo vs. Rest of Brazil

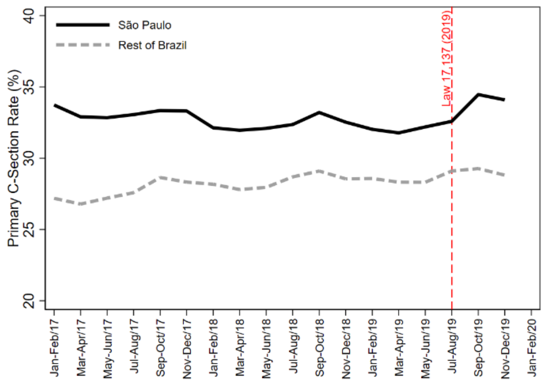

Figure S2: Raw bimonthly time-trend of Primary CS – São Paulo vs. Rest of Brazil

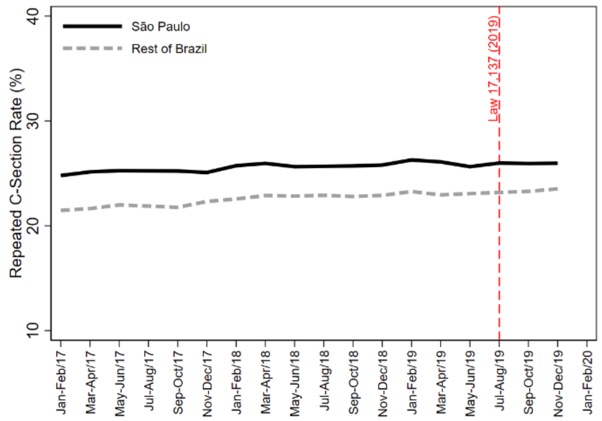

Figure S3: Raw bimonthly time-trend of Repeated CS – São Paulo vs. Rest of Brazil

J. Impact of the law by mother's sociodemographic characteristics

In Figures S4 to S15 below, we present raw trends (left column) and SCM estimates (right column) of mothers' characteristics. The figures show no evidence of compositional changes in the proportion of high-educated (12 years or more) vs. low-educated (11 years of education or fewer) mothers, young (26 years or less) vs. non-young (27 years or more) mothers and married vs. unmarried mothers in São Paulo relative to the rest of Brazil.

However, Figures S5 and S7 suggest that the overall CS rate among low-educated mothers was more affected than that among high-educated mothers. Similarly, Figures S9 and S11 show that the overall CS among young mothers was more responsive to the introduction of the law than that among non-young mothers. Finally, Figures S13 and S15 show that the overall CS rate among unmarried mothers was more responsive to the law than for married mothers. Thus, the SCM estimates for different sociodemographic groups of mothers suggest heterogeneous effects of the law on the overall CS rate.

While the epidemiology literature has documented that the upward trend in CS rates in Brazil during the 2000s has been mainly driven by high-educated, young, and primiparous mothers (Barros et al., 2015), our results suggest that the increase in CS rate with the introduction of the law was driven by low-educated, young, and unmarried mothers. One interpretation of these heterogeneous effects is that while the secular increase in CS rates in Brazil has been driven by high-educated mothers, the effect of the law is likely to be concentrated among a group of mothers where CS rates are comparatively low. In other words, the "compliers" with the law are more likely to be low-educated, young, and unmarried mothers.

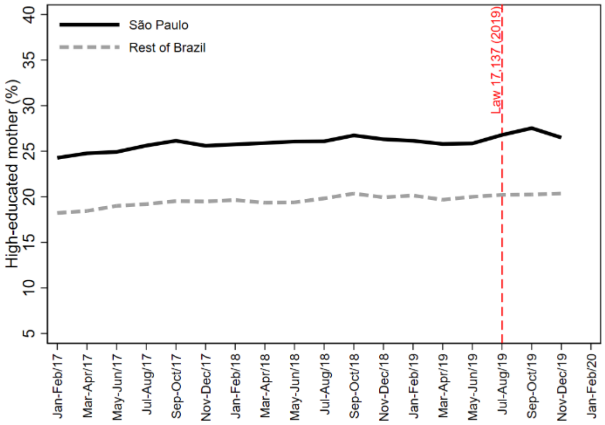

Figure S4: Raw bimonthly time-trend for high-educated mothers' prevalence in São Paulo and for the rest of Brazil

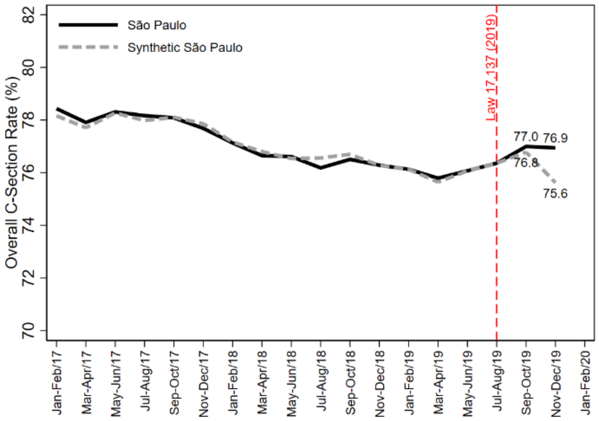

Figure S5: Estimated effect from SCM for the Overall CS rate among high-educated mothers

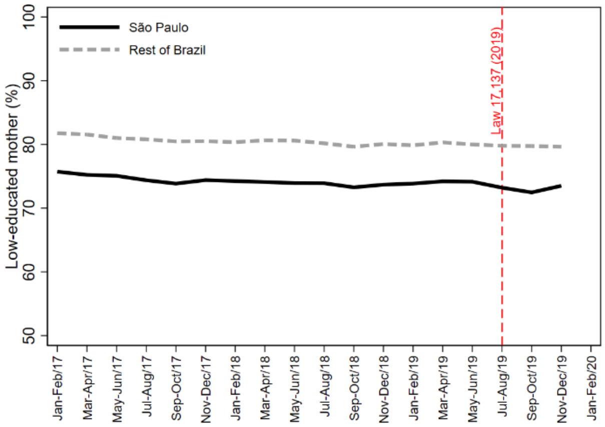

Figure S6: Raw bimonthly time-trend for low-educated mothers' prevalence in São Paulo and for the rest of Brazil

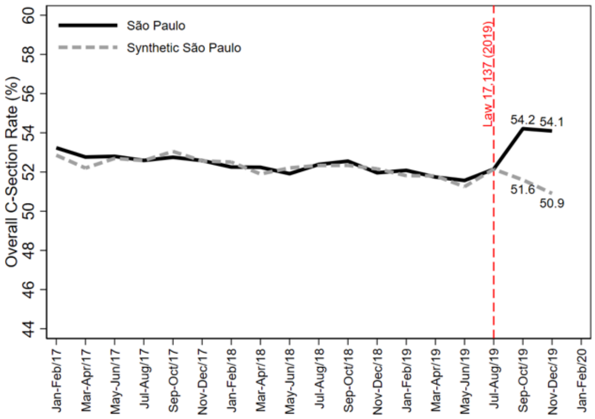

Figure S7: Estimated effect from SCM for the Overall CS rate among low-educated mothers

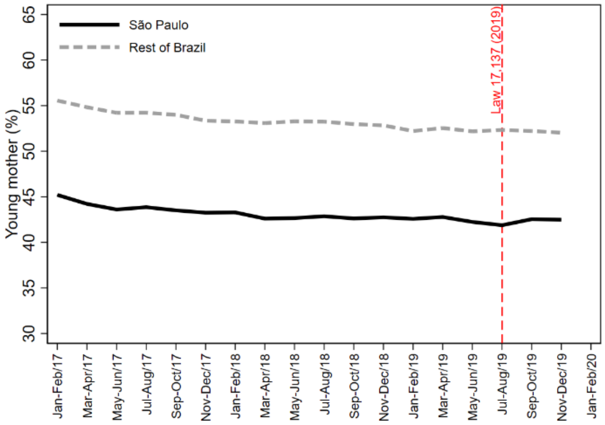

Figure S8: Raw bimonthly time-trend for young mothers' prevalence in São Paulo and for the rest of Brazil

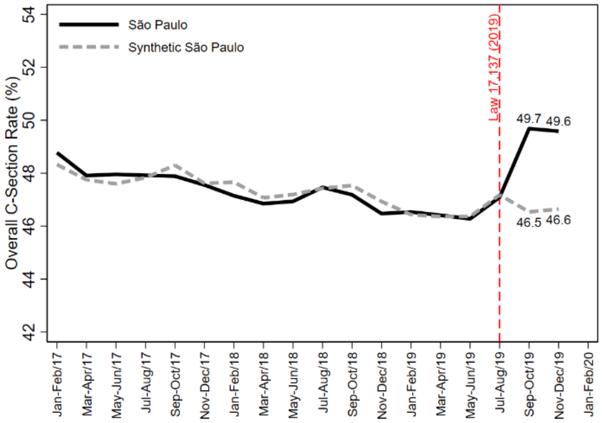

Figure S9: Estimated effect from SCM for the Overall CS rate among young mothers

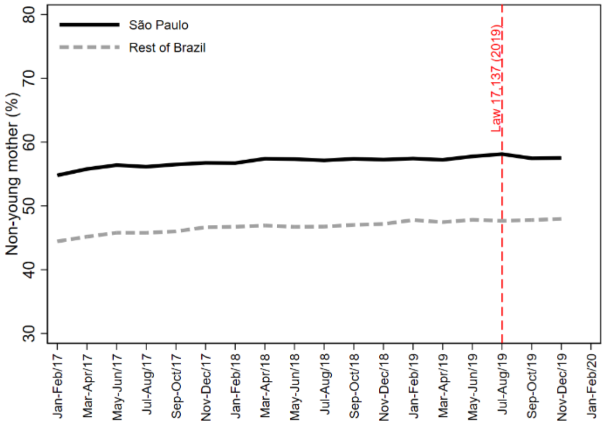

Figure S10: Raw bimonthly time-trend for non-young mothers' prevalence in São Paulo and for the rest of Brazil

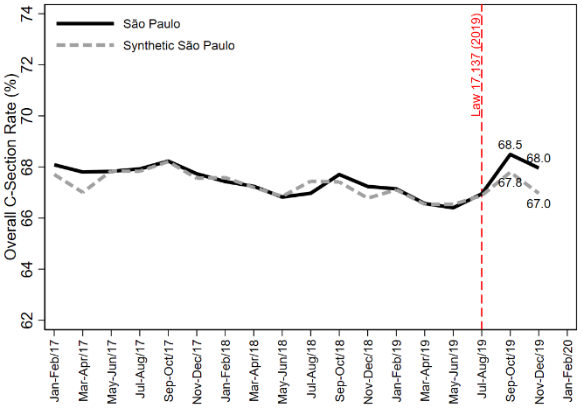

Figure S11: Estimated effect from SCM for the Overall CS rate among non-young mothers

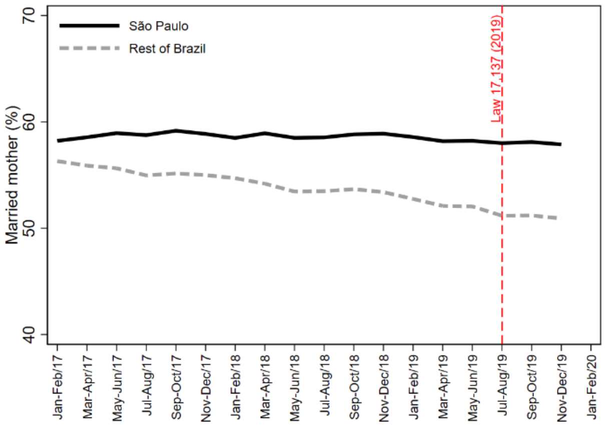

Figure S12: Raw bimonthly time-trend for married mothers' prevalence in São Paulo and for the rest of Brazil

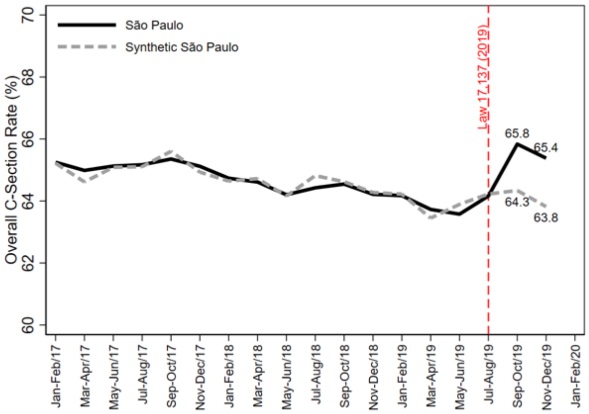

Figure S13: Estimated effect from SCM for the Overall CS rate among married mothers.

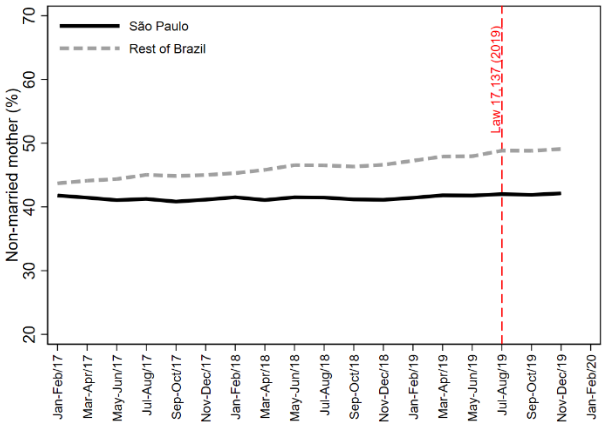

Figure S14: Raw bimonthly time-trend for unmarried mothers' prevalence in São Paulo and for the rest of Brazil.

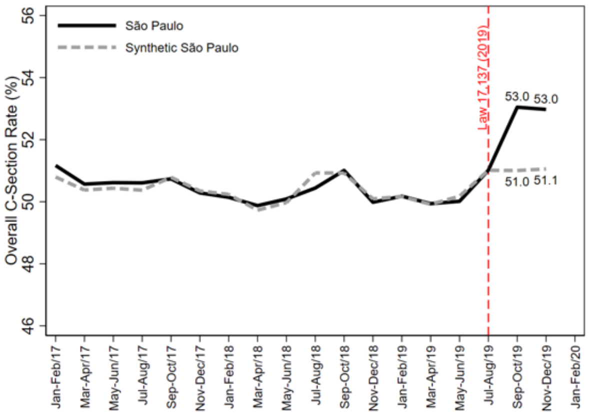

Figure S15: Estimated effect from SCM for the Overall CS rate among unmarried mothers

Finally, our data does not allow us to examine the motivations for requesting a CS. However, in Figures S16 and S17 below we examine whether the increase in CS rates is driven by mothers who had previous vaginal deliveries or those without a delivery history. The figures show that the introduction of the law affects both types of primary CS rates. Among mothers with vaginal delivery history, we find an effect of 0.5pp (or 8.2%) in Sep-Oct/2019, and 0.6pp (or 9.8%) in Nov-Dec/2019. Among mothers without a delivery history, we estimate an increase of 0.9pp (or 3.3%) in Sep-Oct/2019, and 0.7pp (or 2.6%) in Nov-Dec/2019.

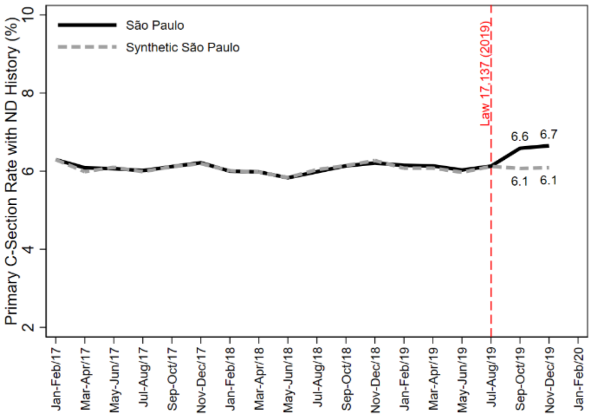

Figure S16: SCM estimate for Primary CS Rate with vaginal delivery history

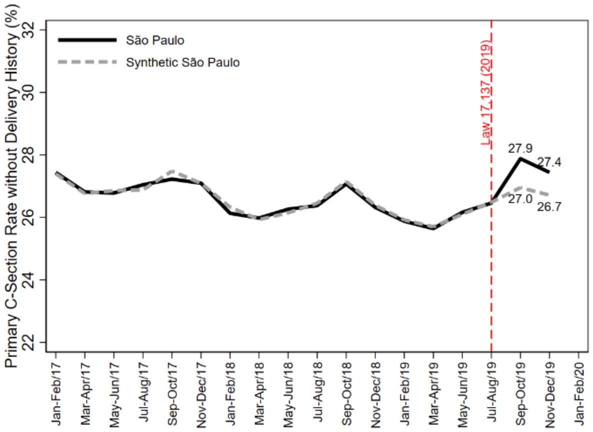

Figure S17: SCM estimate for Primary CS Rate without a delivery history

K. Impact of the law on fertility

In Figure S18, we plot the bimonthly time-trend of live births in São-Paulo and the rest of Brazil. The figure shows no change in the trend of live births in the 4 months after the law was implemented. In Figure S19, we use the SCM to estimate changes in live births. The figure shows that there are no differences in the number of live births between São Paulo and synthetic São Paulo. While our sample ends in December 2019, future work could investigate fertility responses 9 months after the implementation of the law.

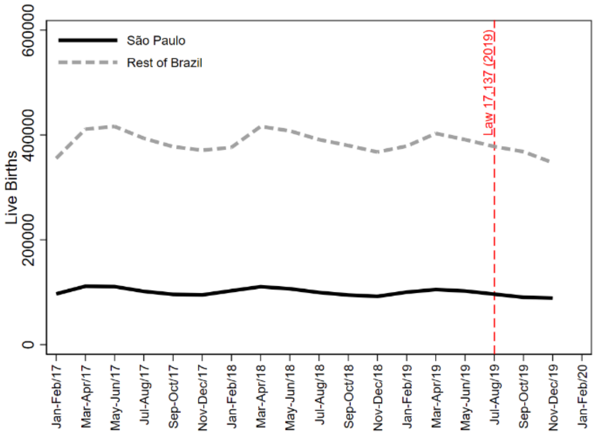

Figure S18: Raw bimonthly time-trend of the number of live births in São Paulo and rest of Brazil

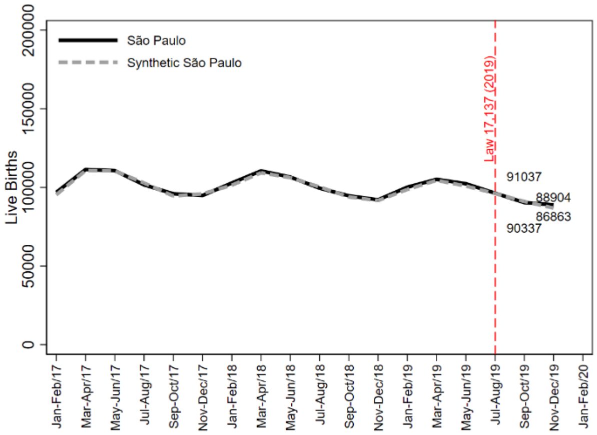

Figure S19: Estimated effect from SCM for the number of live births

L. Impact of the law by availability of obstetricians and surgical obstetric beds

To investigate the role of supply factors, we compare the effects of the law across different health regions in São Paulo. We measure the availability of obstetricians and surgical obstetric beds at the health region level within São Paulo state. We use the number of obstetricians and surgical obstetric beds (Source: Cadastro Nacional de Estabelecimentos de Saúde/Datasus) as

proxies for the availability of human and physical resources to perform C-sections. These variables are available for the 17 health regions of São Paulo state, clusters of municipalities by which the healthcare service is organized and coordinated by the State Government.

First, for each health region in São Paulo state, we normalize the number of obstetricians and surgical obstetric beds by the total number of live births (per 1,000 live births) and compute the median for both measures in 2019. Second, we split the São Paulo sample into two groups: newborns from areas with high (above 9.5) vs. low (9.5 or below) availability of obstetricians, as well as those from areas with a high (above 8.9) vs. low (8.9 or below) availability of surgical obstetric beds. Third, we employ the SCM and estimate the effect of the introduction of the Law 17,137 for these two groups.

Figures S20 to S23 below demonstrate that the effects of the law on CS rates are similar in areas with below- and above-median availability of obstetricians and surgical obstetric beds. This suggests a limited role of supply-side factors on the effect of the law.

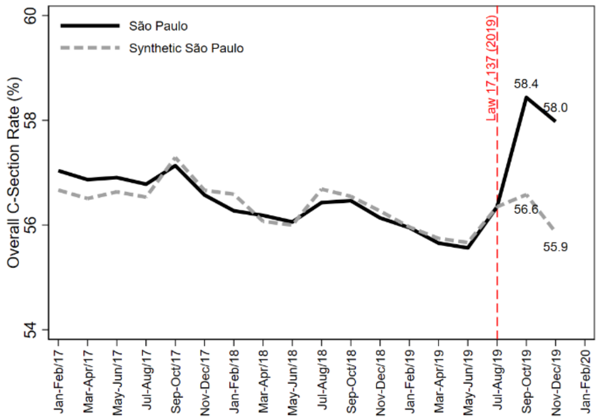

Figure S20: SCM estimate for Overall CS Rate for Areas of São Paulo with Above-Median Availability of Obstetricians

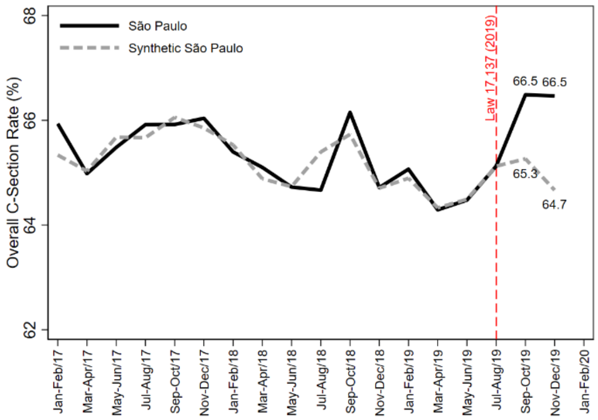

Figure S21: SCM estimate for Overall CS Rate for Areas of São Paulo with Below-Median Availability of Obstetricians

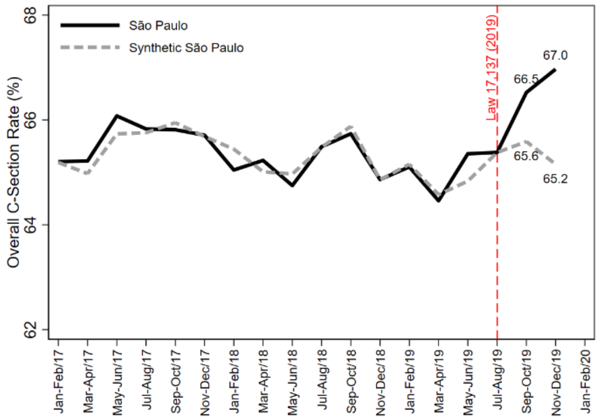

Figure S22: SCM estimate for Overall CS Rate for Areas of São Paulo with Above-Median Availability of Surgical Obstetric Beds

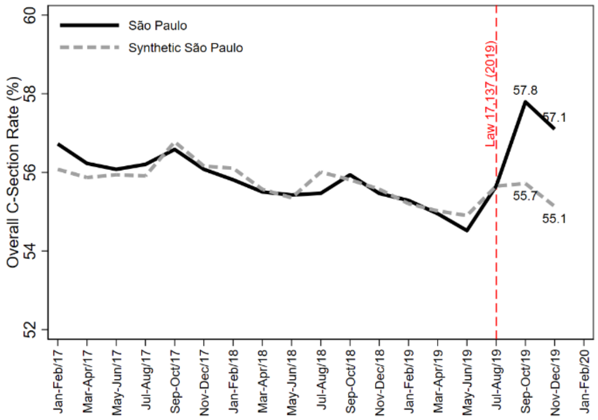

Figure S23: SCM estimate for Overall CS Rate for Areas of São Paulo with Below-Median Availability of Surgical Obstetric Beds

While not reported here, it is worth mentioning that the law did not affect the availability of obstetricians or surgical obstetric beds (results available from the authors upon request).

M. Impact of the law on health at birth

In the research letter we did not present results on the effect of the law on health outcomes at birth because these estimates are likely to be confounded by compositional bias. Since the law stipulates that C-sections are permitted after 39 weeks of gestation, the law is expected to mechanically increase gestational length amongst those deliveries via C-section. Therefore, birth outcomes can only be examined conditional on gestational length, resulting in a selected sample of babies being analysed. Indeed, gestational length is one of the outcomes used in studies looking at impacts on health at birth.

Despite these limitations, in Figures S24 to S27 below, we present both raw trends (left column) and SCM estimates (right column) of a commonly used measure of health at birth, birth weight (Clarke et al., 2021). We are unable to detect any effects of the law on average birth weight or the prevalence of low birth weight (<2500 g) births.

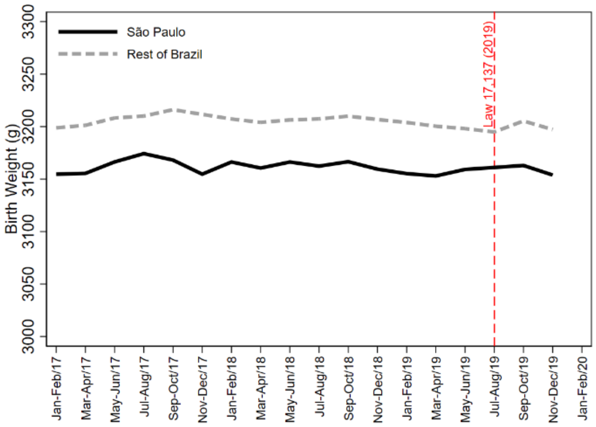

Figure S24: Raw bimonthly time-trend of birth weight (g) in São Paulo and for the rest of Brazil

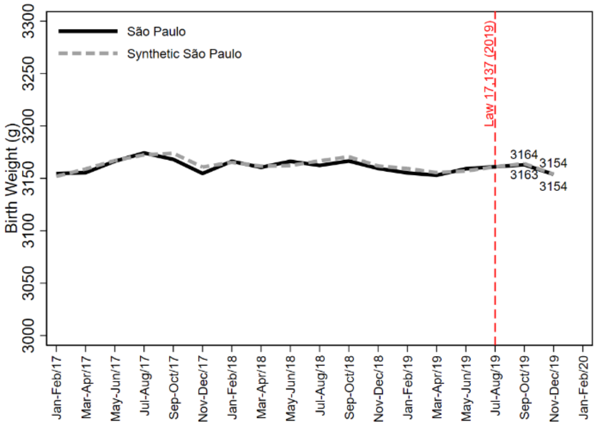

Figure S25: Estimated effect from SCM for birth weight

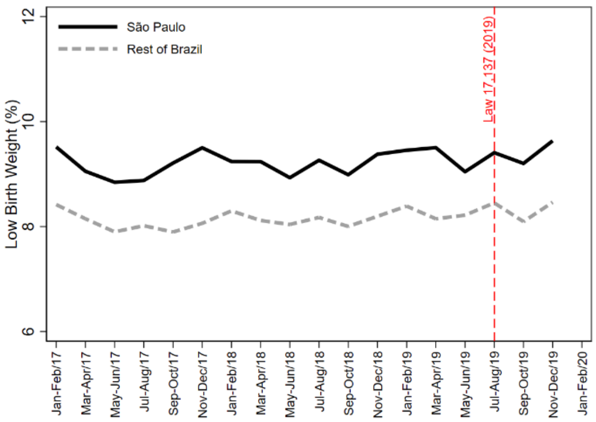

Figure S26 Raw bimonthly time-trend for the prevalence low birth weight babies in São Paulo and for the rest of Brazil

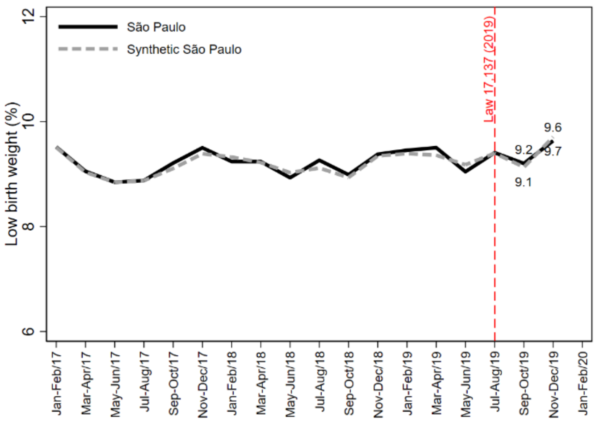

Figure S27: Estimated effect from SCM for the prevalence of low-birth weight babies

N. SCM estimates using a placebo policy date

Figures S28 to S30 use data for 2016-2018 (rather than 2017-2019 in the research letter) to implement the SCM method. Assuming a placebo policy change in July/August 2018 (rather than July/August 2019), we do not find any substantial differences between São Paulo and Synthetic São Paulo. This confirms the absence of any placebo effects, reinforcing the validity of our identification strategy.

Figure S28. Estimated placebo effect on Overall C-section rates

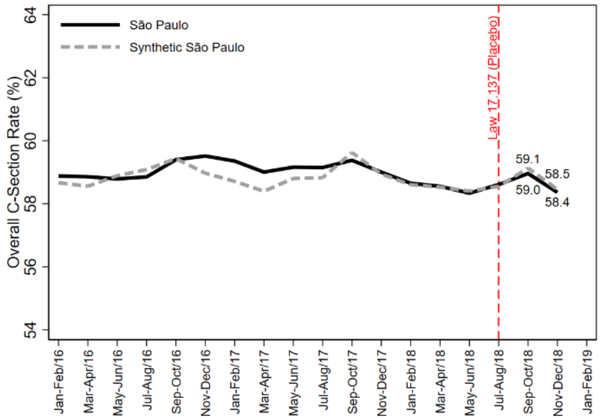

Figure S29. Estimated placebo effect on Primary C-section rates

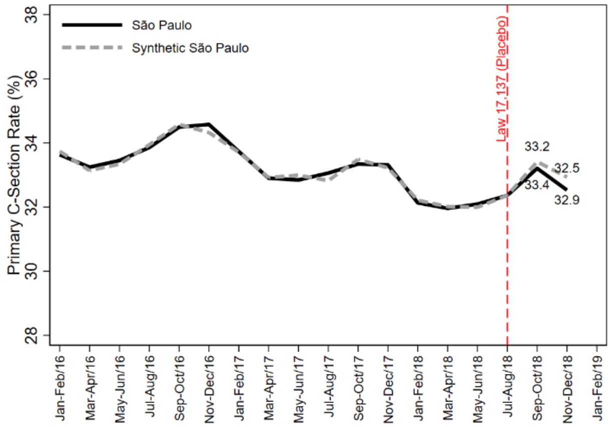

Figure S30. Estimated placebo effect on Primary C-section rates

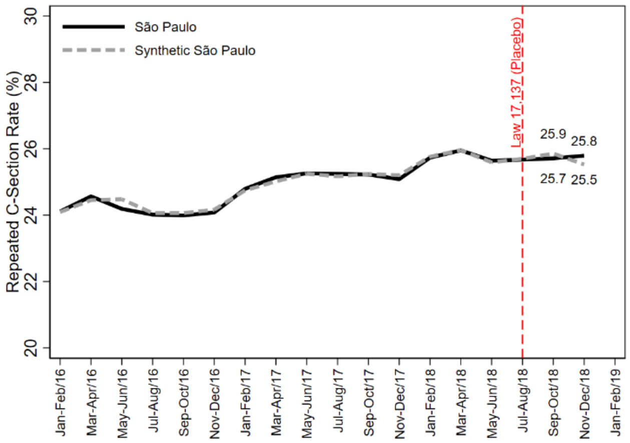

O. Excluding neighbouring states

To assess the validity of our analysis to potential migration biases from neighbouring states, Figures S31 to S33 replicate the SCM analysis displayed in Figure 2 (a, b, c) after excluding the four states that share borders with São Paulo: Mato Grosso do Sul, Minas Gerais, Paraná, and Rio de Janeiro. Our findings are virtually the same.

Figure S31. Estimated placebo effect on Overall C-section rates

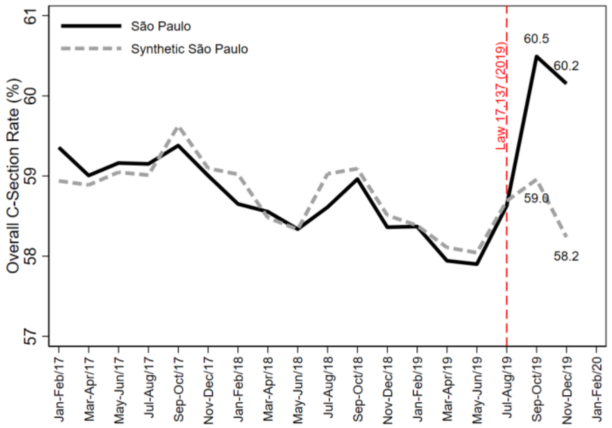

Figure S32. Estimated placebo effect on Primary C-section rates

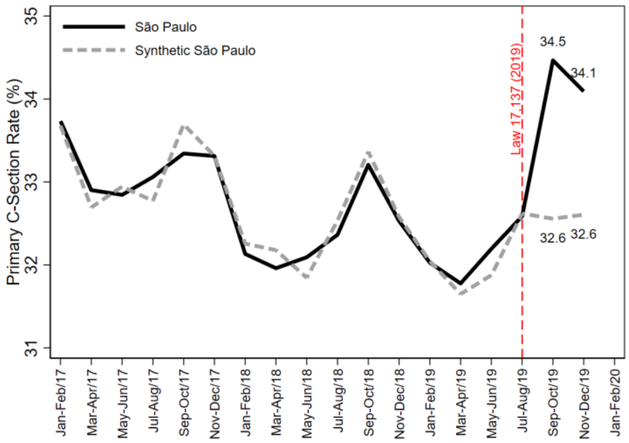

Figure S33. Estimated placebo effect on Primary C-section rates

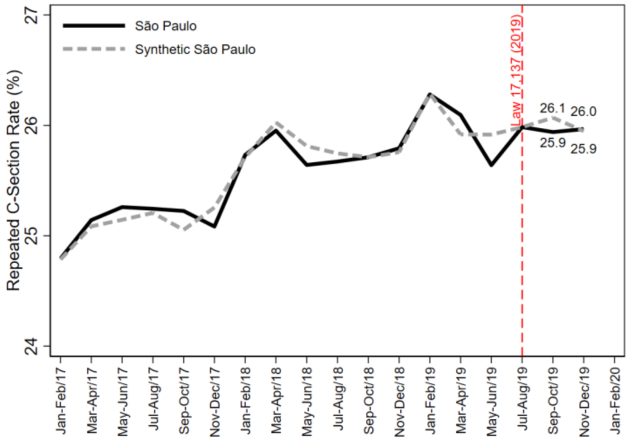

P. Summary of quasi-experimental studies

Table S4 provides a summary of quasi-experimental studies of other factors affecting CS rates:

Table S4: Summary of quasi-experimental estimates of other factors affecting CS rates

Author (Year)	Country	Factor	Finding
Mühlrad (2022)	Sweden	Information shock: dissemination of new scientific evidence suggesting that planned C-section is the preferred delivery method for singleton breech births at term.	24% higher likelihood of planned C-section delivery for breech births.
Foo et al. (2017)	US	Supply-side financial incentives: Change in the price differential for C-sections vs vaginal deliveries paid by commercial insurers to	Increasing the physician price differential by 1 standard deviation ($420) leads to a 12% increase in the odds ratio for C-

		hospitals and physicians.	section delivery.<br>Increasing the hospital price differential by one standard deviation ($5,805) for births delivered by hospital-exclusive physician groups yields a 31 percent increase in the odds ratio.
Borra et al. (2019)	Spain	Demand-side financial incentives: Removal of<br>€2,500 baby-bonus for babies born starting after 1 Jan 2011.	Increased in number of daily C-sections in late December 2010 (about 120 per day extra when focusing on the one-week window).
de Elejalde & Giolito (2021)	Chile	Supply-side & demand-side incentives: a policy change that increased delivery at private hospitals by reducing the out-of-pocket cost for women with public insurance (private hospitals receive the same price for a vaginal or C-section delivery).	Increased the probability of a C-section by 8.6pp.
Currie & MacLeod (2008)	US	Supply-side accountability: adoption of tort reforms by states in the US during the 1980s and 1990s.	The adoption of the rule of joint and several liability (the so-called deep pockets rule), in which doctors are held more accountable for their own actions, reduced the probability of C-section by 7%.
Amaral-Garcia et al. (2021)	England	Effect of internet diffusion on C-section deliveries.	First-time mothers living in areas with better internet access are 6% more likely to obtain a C-section. No corresponding effects on healthcare outcomes.

References

Abadie A. 2021. Using Synthetic Controls: Feasibility, Data Requirements, and Methodological Aspects. *Journal of Economic Literature*. 59(2):391–425

Amaral-Garcia S, Nardotto M, Propper C, Valletti T. 2021. Mums Go Online: Is the Internet Changing the Demand for Healthcare? *The Review of Economics and Statistics*. 1–45

Barros FC, Matijasevich A, Maranhão AGK, Escalante JJ, Rabello Neto DL, et al. 2015. Cesarean sections in Brazil: will they ever stop increasing? *Revista Panamericana de Salud Pública*. 38:217–25

Borra C, González L, Sevilla A. 2019. The Impact of Scheduling Birth Early on Infant Health. *Journal of the European Economic Association*. 17(1):30–78

Clarke D, Oreffice S, Quintana-Domeque C. 2021. On the Value of Birth Weight. *Oxford Bulletin of Economics and Statistics*

Currie J, MacLeod WB. 2008. First Do No Harm? Tort Reform and Birth Outcomes*. *The Quarterly Journal of Economics*. 123(2):795–830

de Azevedo Bittencourt SD, Domingues RMSM, da Costa Reis LG, Ramos MM, do Carmo Leal M. 2016. Adequacy of public maternal care services in Brazil. *Reproductive health*. 13(3):257–65

de Elejalde R, Giolito E. 2021. A demand-smoothing incentive for cesarean deliveries. *Journal of Health Economics*. 75:102411

de Oliveira VH, Lee I, Quintana-Domeque C. 2021. Natural Disasters and Early Human Development: Hurricane Catarina and Infant Health in Brazil. *Journal of Human Resources*. 0816-8144R1

Entringer AP, Pinto M, Dias MAB, Gomes MA de SM. 2018. Cost-effectiveness analysis of spontaneous vaginal delivery and elective cesarean for normal risk pregnant women in the Brazilian Unified National Health System. *Cadernos de saude publica*. 34:

Ferman B, Pinto C, Possebom V. 2020. Cherry Picking with Synthetic Controls. *Journal of Policy Analysis and Management*. 39(2):510–32

Foo PK, Lee RS, Fong K. 2017. Physician prices, hospital prices, and treatment choice in labor and delivery. *American Journal of Health Economics*. 3(3):422–53

Leal M do C, Esteves-Pereira AP, Viellas EF, Domingues RMSM, Gama SGN da. 2020. Prenatal care in the Brazilian public health services. *Revista de saúde pública*. 54:

Leal M do C, Pereira APE, Domingues RMSM, Filha MMT, Dias MAB, et al. 2014. Obstetric interventions during labor and childbirth in Brazilian low-risk women. *Cadernos de saude publica*. 30:S17–32

Massuda A, Hone T, Leles FAG, de Castro MC, Atun R. 2018. The Brazilian health system at crossroads: progress, crisis and resilience. *BMJ global health*. 3(4):e000829

Mühlrad H. 2022. Cesarean sections for high-risk births: health, fertility, and labor market outcomes. *The Scandinavian Journal of Economics*.
